# Effects of Leg Motor Imagery Combined With Electrical Stimulation on Plasticity of Corticospinal Excitability and Spinal Reciprocal Inhibition

**DOI:** 10.3389/fnins.2019.00149

**Published:** 2019-02-21

**Authors:** Yoko Takahashi, Michiyuki Kawakami, Tomofumi Yamaguchi, Yusuke Idogawa, Shigeo Tanabe, Kunitsugu Kondo, Meigen Liu

**Affiliations:** ^1^Department of Rehabilitation Medicine, Keio University School of Medicine, Tokyo, Japan; ^2^Tokyo Bay Rehabilitation Hospital, Chiba, Japan; ^3^Department of Physical Therapy, Yamagata Prefectural University of Health Sciences, Yamagata, Japan; ^4^Faculty of Rehabilitation, School of Health Sciences, Fujita Health University, Toyoake, Japan

**Keywords:** motor imagery, motor-evoked potential, H-reflex, disynaptic reciprocal inhibition, peripheral nerve electrical stimulation

## Abstract

Motor imagery (MI) combined with electrical stimulation (ES) enhances upper-limb corticospinal excitability. However, its after-effects on both lower limb corticospinal excitability and spinal reciprocal inhibition remain unknown. We aimed to investigate the effects of MI combined with peripheral nerve ES (MI + ES) on the plasticity of lower limb corticospinal excitability and spinal reciprocal inhibition. Seventeen healthy individuals performed the following three tasks on different days, in a random order: (1) MI alone; (2) ES alone; and (3) MI + ES. The MI task consisted of repetitive right ankle dorsiflexion for 20 min. ES was percutaneously applied to the common peroneal nerve at a frequency of 100 Hz and intensity of 120% of the sensory threshold of the tibialis anterior (TA) muscle. We examined changes in motor-evoked potential (MEP) of the TA (task-related muscle) and soleus muscle (SOL; task-unrelated muscle). We also examined disynaptic reciprocal inhibition before, immediately after, and 10, 20, and 30 min after the task. MI + ES significantly increased TA MEPs immediately and 10 min after the task compared with baseline, but did not change the task-unrelated muscle (SOL) MEPs. MI + ES resulted in a significant increase in the magnitude of reciprocal inhibition immediately and 10 min after the task compared with baseline. MI and ES alone did not affect TA MEPs or reciprocal inhibition. MI combined with ES is effective in inducing plastic changes in lower limb corticospinal excitability and reciprocal Ia inhibition.

## Introduction

Motor imagery (MI) has been described as a dynamic state during which the representation of a given motor act is internally rehearsed within working memory without any overt motor output (Decety and Grezes, 1999). Brain activation during MI is similar to that observed during motor execution (Jackson et al., 2001; Chen et al., 2016; Sacheli et al., 2017). MI has been shown to increase corticospinal excitability, the H-reflex, spinal stretch reflex, and reciprocal inhibition (Kiers et al., 1997; Hale et al., 2003; Bakker et al., 2008; Aoyama and Kaneko, 2011; Lebon et al., 2012; Kato and Kanosue, 2017; Ruffino et al., 2017; Kawakami et al., 2018). Mental practice using MI is widely used in sports and rehabilitation (Jackson et al., 2001; de Lange et al., 2008; Malouin and Richards, 2010; Mrachacz-Kersting et al., 2012, 2016; Grospretre et al., 2016; Guerra et al., 2017; Ruffino et al., 2017).

Introducing the plasticity of neural circuits involved in motor activation is important for motor recovery (Stefan et al., 2000; Wolpaw and Tennissen, 2001; Wolpaw, 2007; Di Pino et al., 2014). Previous studies using a paired associative stimulation (PAS) protocol (Stefan et al., 2000; Stinear and Hornby, 2005) have shown that spike-timing-dependent input to the motor cortex and peripheral nerves is important for the induction of plasticity in the motor cortex. Input to the motor cortex and spinal cord is also important for inducing plastic changes in spinal circuits (Wolpaw and Tennissen, 2001; Chen et al., 2016; Yamaguchi et al., 2018). One previous study used MI as the input to the motor cortex and ES as the input to the peripheral nerve (Saito et al., 2013). Indeed, a combination of finger movement MI and ES was shown to increase corticospinal excitability to a greater extent than MI or ES alone (Saito et al., 2013). Finger movement MI during action observation combined with ES for 20 min induced plastic change of corticospinal excitability at the time just after the end of the intervention, but each intervention alone was ineffective (Yasui et al., 2018). In the lower limb, Mrachacz-Kersting et al. (2012) showed that the corticospinal excitability during the application of MI using electroencephalography combined with peripheral nerve ES was greater than that during the period without MI. However, whether these effects remain after the task is currently unknown. Systematic reviews have reported the effects of MI on the upper or lower limb hemiparesis in patients with stroke (Braun et al., 2006; Guerra et al., 2017). The number of studies in the lower limb were less than those in upper limb; therefore, the therapeutic effect has not been established. Basic knowledge on the neurological effects of MI and MI combined with ES are needed to develop therapeutic strategies for lower limb MI.

The neural control of the upper and lower limb is differ depending on the task (Arya and Pandian, 2014). For motor control of the lower limb (such as that required for walking), both spinal and descending neural circuits from the motor cortex are important. Spinal reciprocal inhibition between agonist and antagonist muscles is responsible for the achievement of smooth movements (Crone and Nielsen, 1989; Morita et al., 2001). Reciprocal inhibition of calf muscles is often reduced or facilitated in patients with stroke and spinal cord injury (Crone and Nielsen, 1994; Okuma et al., 2002; Crone et al., 2003). Normalization of the reciprocal inhibition is as important as that of the neural circuits in the brain for motor recovery in the lower limb or for gait rehabilitation (Dietz and Sinkjaer, 2007).

In this study, we aimed to examine the effects of MI combined with ES on the plasticity of both corticospinal excitability and spinal reciprocal inhibition in the lower limb. We hypothesized that MI combined with peripheral nerve electrical stimulation (ES) may be superior in inducing plastic changes in cortical and spinal neural circuits than MI or ES alone.

## Materials and Methods

The Tokyo Bay Rehabilitation Hospital ethics committee approved the study protocol (approval number: 175-2). All tests were performed at the Tokyo Bay Rehabilitation Hospital. This study was registered in the University Hospital Medical Information Network (UMIN; registration number: 000028087). All participants provided written informed consent prior to enrolment. The procedures complied with the Declaration of Helsinki.

Seventeen healthy adults (mean age: 24.6 ± 2.1 years, seven females) participated in this study. None of the participants had history of neurological disease or was receiving any acute or chronic medications that could affect the central nervous system.

**FIGURE 1:**
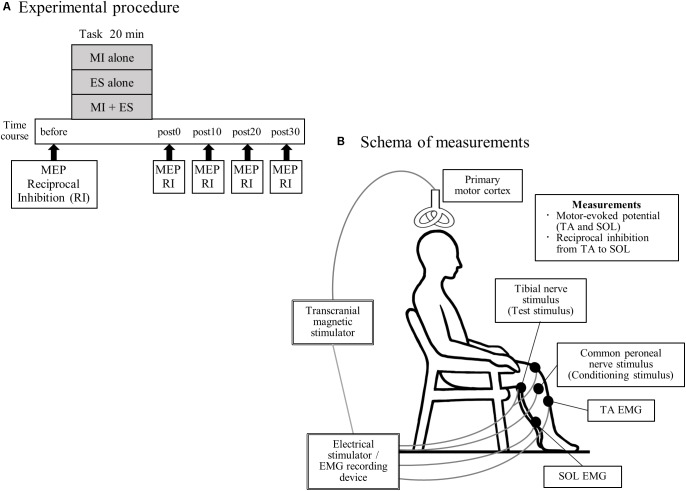
Experimental procedure and schema of measurements. **(A)** Seventeen healthy individuals performed the following three tasks, on separate days in a random order: (1) motor imagery (MI) alone; (2) peripheral nerve electrical stimulation (ES) alone; and (3) MI combined with ES (MI + ES). We measured motor-evoked potentials (MEPs) of the tibialis anterior (TA) and soleus (SOL) muscles and reciprocal inhibition (RI) at baseline (before), immediately after (post0), 10 min after (post10), 20 min after (post20), and 30 min after (post30) the task. **(B)** Participants remained seated on a chair with a backrest, in a relaxed position. During MEP measurement, we stimulated the left primary motor cortex of the leg area using a transcranial magnetic stimulator and recorded electromyography (EMG) of the right TA and SOL. Reciprocal inhibition was assessed using a soleus H-reflex conditioning-test paradigm. The H-reflex in SOL was elicited by stimulating the tibial nerve at the popliteal fossa. A positive stimulation electrode was set above the patella. The conditioning stimulus was applied to the common peroneal nerve below the fibular head.

All participants performed the following three tasks, in a random order: (1) MI alone; (2) peripheral nerve ES alone (ES alone); and (3) MI combined with ES (MI + ES; Figure 1A). Tasks were performed on different days, and were separated by more than 7 days to minimize carry-over effects. Participants performed each task for 20 min. Motor-evoked potential (MEP) and disynaptic reciprocal inhibition were assessed before, immediately after (post0), and 10 (post10), 20 (post20), and 30 min (post30) after the task.

During the experimental sessions, participants remained seated on a chair with a backrest in a relaxed position with 80° hip flexion, 80° knee flexion, 10° ankle plantar flexion and their feet on the floor (Figure 1B).

### Task

#### Motor Imagery

Participants were instructed to imagine dorsiflexion of their right ankle with the help of a video, for 20 min (kinesthetic motor imaging of ankle dorsiflexion). A trial consisted of imagination for 2 s and a rest period of 4 s. The full task consisted of 200 trials that were repeated over a 20 min period. To avoid muscle contraction during the MI, we monitored the electromyographic recording of the tibialis anterior (TA) and soleus (SOL) muscles and provided verbal feedback to avoid muscle contraction when signs of muscle contraction were identified.

#### Peripheral Nerve Electrical Stimulation

Electrical stimulation was applied to the common peroneal nerve at the fibular head. ES was delivered with a frequency of 100 Hz (pulse width 1 ms) for 2 s at an intensity of 120% of the sensory threshold of the TA at rest without muscle contraction. This stimulus intensity was determined so that MEP can be increased to a level greater than those at rest when combined with MI (Yamaguchi et al., 2012). A trial consisted of stimulation for 2 s and a rest period of 4 s. 200 trials were conducted over 20 min.

#### Motor Imagery Combined With Electrical Stimulation

MI was applied in the same manner as in the MI alone paradigm. Participants were asked to imagine dorsiflexion of their right ankle at the same time that ES was applied. 200 trials were conducted over 20 min.

### Assessment

#### Motor-Evoked Potential

The schema of the experimental measurements is presented in Figure 1B. MEPs were assessed using transcranial magnetic stimulation. Since the MI task consisted of ankle dorsiflexion, we selected the TA as a task-related muscle, and the SOL as a task-unrelated muscle. We stimulated the left primary motor cortex using a Magstim 200 magnetic stimulator (The Magstim Company, Whitland, Dyfed, United Kingdom) and a double-cone coil. The stimulation site was selected to coincide with the point where MEP amplitude of the right TA was the largest. The stimulus intensity was defined as 120% of the resting motor threshold of each muscle. The resting motor threshold was defined as the intensity that evoked responses of 100-μV (at 5 of 10) in the TA or SOL muscle at rest. A total of 15 MEPs were recorded for each muscle. Peak-to-peak amplitudes were averaged for each time point.

#### Reciprocal Inhibition

We measured spinal reciprocal inhibition of the calf muscles, i.e., reciprocal inhibition from the TA to the SOL. Reciprocal inhibition was assessed using a soleus H-reflex conditioning-test paradigm. The H-reflex in the soleus muscle was elicited by stimulating the tibial nerve at the popliteal fossa (1 ms rectangular pulse). A positive (anode) stimulation electrode was set above the patella. Throughout the experiment, the test H-reflex amplitude was maintained at 15–20% of the amplitude of the maximum motor response for the SOL, as previously described (Crone et al., 1990). The conditioning stimulus was delivered to the common peroneal nerve using surface electrodes positioned below the fibular head. The conditioning stimulus intensity was defined as 1.0 × motor threshold. The motor threshold was defined as the intensity that evoked a response of 100 μV in the TA at rest. The common peroneal nerve-stimulating electrode was carefully positioned to avoid activation of the peroneus muscles, thereby ensuring selective stimulation of the deep branch of the peroneal nerve (Crone et al., 1990). The conditioning stimulus was repeatedly checked during the experiments by monitoring the M wave of the TA. The interval between the conditioning and test stimulus was set at 0, 1, 2, and 3 ms (Fujiwara et al., 2011). The optimal interval to produce disynaptic reciprocal inhibition by stimulating the common peroneal nerve was determined at the beginning of each session and used throughout. Ten conditioned and 10 test H-reflexes were recorded at each time point. Reciprocal inhibition was defined as the mean conditioned H-reflex amplitude/mean test H-reflex amplitude.

#### Motor Imagery Ability

To assess MI ability, all participants answered the Vividness of Movement Imagery Questionnaire-2 (VMIQ-2, Roberts et al., 2008) at the beginning of the experiment. The VMIQ-2 is one of the most commonly used questionnaires to assess the vividness of MI. This questionnaire is used to determine the level of vividness with which the 12 motor tasks can be imagined from the viewpoint of the following three factors: internal visual imagery, external visual imagery, and kinesthetic imagery. Subjects rate the vividness of their MI on a five-point scale (1 = perfectly vivid and as clear as normal vision, 5 = no image at all). The maximum score is 60 points per factor. A low score indicates greater imagery ability.

### Statistical Analysis

We calculated the sample size of the study based on our previous study that examined the after-effects of reciprocal inhibition by ES combined with voluntary contraction (Takahashi et al., 2017). We performed the power analysis based on the following: effect size, 1.39; α error, 0.05; and statistical power, 0.95 using G^∗^Power 3.1.9.2 for Windows (Heinrich Heine University, Dusseldorf, Germany). The power analysis indicated that 12 samples were needed. We recruited 17 participants, which fully satisfied the power sample size requirements.

A two-factor repeated-measures analysis of variance (ANOVA) was used to analyze the main and interaction effects of task (MI alone, ES alone, and MI + ES) and time (before, post0, post10, post20, and post30) on TA MEP amplitudes, test H amplitudes, and reciprocal inhibition. Paired *t*-tests, with Bonferroni correction for multiple comparisons, were performed as post-hoc tests, when a significant result was obtained in the primary analyses. Prior to ANOVA, we confirmed that all TA MEPs and reciprocal inhibition variables were normally distributed using Shapiro–Wilk tests. SOL MEPs were not normally distributed; therefore, we used Wilcoxon signed rank tests to compare SOL MEPs before and at each time point after the task.

To assess the relationship between MI ability and the effects of the MI + ES task, a Spearman’s rank correlation coefficient was performed. We compared the correlation between the VMIQ-2 mean score (internal visual imagery, external visual imagery, and kinesthetic imagery) and changes in TA MEPs and reciprocal inhibition after the intervention. Changes in TA MEPs and reciprocal inhibition after the intervention were calculated as the difference between the valuables before and post0.

Statistical analyses were performed using SPSS 24 (IBM Corp., Armonk, NY, United States) for Windows. Results with *p* < 0.05 were considered statistically significant.

## Results

### MEP Amplitudes

**FIGURE 2:**
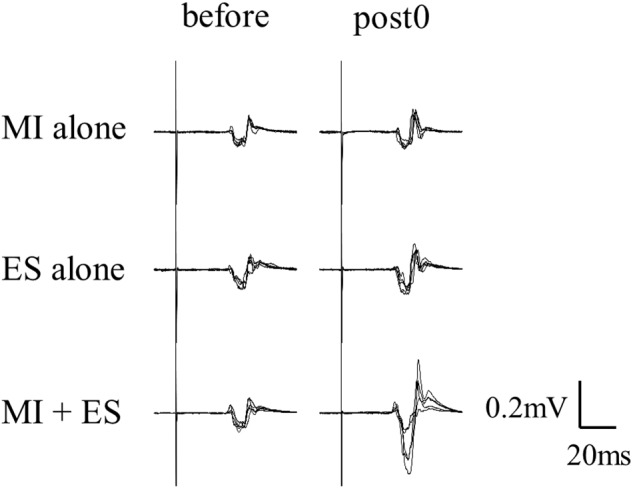
Representative TA MEPs (before and at post0). MI alone and ES alone showed no significant change in TA MEPs at post0 in comparison with baseline. TA MEP of MI combined with ES (MI + ES) at post0 was significantly higher than at baseline.

**FIGURE 3:**
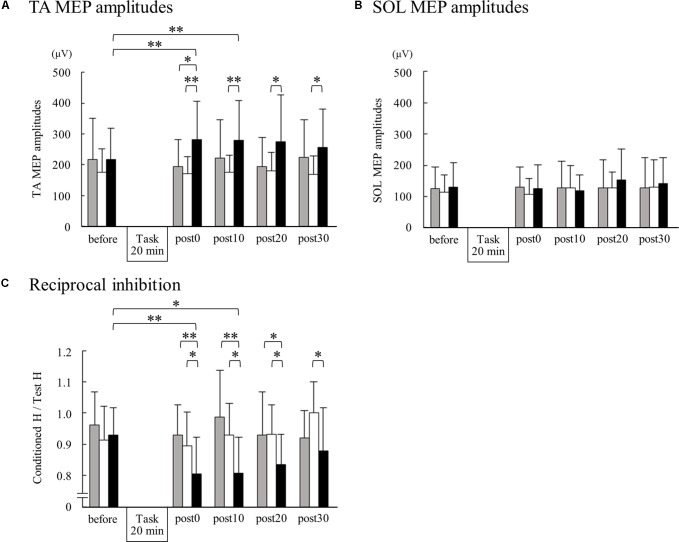
Changes in TA MEPs, soleus (SOL) MEPs, and reciprocal inhibition before and after motor imagery alone, electrical stimulation alone, and motor imagery combined with electrical stimulation. The peak-to-peak amplitudes from 15 TA MEPs **(A)** and 15 SOL MEPs **(B)** measurements acquired at each time point were averaged for each subject. For reciprocal inhibition **(C)**, 10 test and 10 conditioned reflexes were averaged at each time point for each subject. Group means + standard deviations are plotted for each time point; motor imagery alone (gray bar), electrical stimulation alone (white bar), motor imagery combined with electrical stimulation (black bar). Asterisks indicate significant differences as identified by *post hoc* paired *t*-tests (^∗^*p* < 0.05, ^∗∗^*p* < 0.01).

Figure 2 shows the representative change in TA MEPs in one participant. We found a significant main effect of task (*F*_2,32_ = 7.733, *p* = 0.002) and a significant interaction between task (MI alone, ES alone, and MI + ES) and time (before, post0, post10, post20, and post30) (*F*_8,128_ = 2.892, *p* = 0.005) for MEPs of the task-related TA muscle. No significant main effect of time (*F*_4,64_ = 0.854, *p* = 0.497) was observed. *Post hoc* paired *t*-test comparisons revealed that TA MEPs were significantly higher at post0 and post10 after MI + ES than at baseline (post0: *p* = 0.009, post10: *p* = 0.009; Table 1 and Figure 3A). MI alone and ES alone did not produce any significant changes in TA MEPs at any time point. TA MEPs of MI + ES were significantly higher than that of MI alone at post0 (*p* = 0.014). TA MEPs of MI + ES were also significantly higher than that of ES alone at post0 (*p* = 0.002), post10 (*p* = 0.004), post20 (*p* = 0.027), and post30 (*p* = 0.011).

**Table 1:** Statistical results of the *post hoc* paired *t*-tests with Bonferroni correction in tibialis anterior motor-evoked potentials.

Comparison with the baseline within task				
Task	*p*-value [95% confidence interval of mean difference]			
	post0	post10	post20	post30
MI alone	1.0 [-44.3–91.8]	1.0 [-61.1–50.5]	1.0 [-63.9–110.8]	1.0 [-97.1–81.7]
ES alone	1.0 [-75.0–77.6]	1.0 [-65.9–64.2]	1.0 [-81.4–71.9]	1.0 [-68.4–82.1]
MI + ES	0.009^∗∗^ [-117.3–-13.2]	0.009^∗∗^ [-111.6–-12.3]	0.132 [-123.8–9.6]	1.0 [-111.9–35.3]

**Comparison of the value among tasks at the same time points**				
**Task**	***p*-value [95% confidence interval of mean difference]**			
	**post0**	**post10**	**post20**	**post30**

MI alone vs. ES alone	0.819 [-29.1–72.1]	0.245 [-20.6–114.3]	1.0 [-35.9–64.4]	0.213 [-21.7–135.9]
MI alone vs. MI + ES	0.014^∗^ [-162.1–-16.3]	0.152 [-128.9–15.1]	0.052 [-0.6–162.2]	0.947 [-48.7–110.3]
ES alone vs. MI + ES	0.002^∗^ [-180.4–-41.0]	0.004^∗^ [-174.5–-33.1]	0.027^∗^ [-180.8–-9.3]	0.011^∗^ [-157.2–-18.5]


*MI, motor imagery; ES, electrical stimulation; MI + ES, motor imagery combined with electrical stimulation; ^∗∗^p < 0.01, ^∗^p < 0.05.*

We could only measure SOL MEPs in 12 of the 17 participants. A Wilcoxon signed rank test revealed no significant difference among SOL MEPs at any time point (Figure 3B).

### Reciprocal Inhibition

No significant main effects of task (*F*_2,32_ = 0.339, *p* = 0.715) or time (*F*_4,64_ = 0.971, *p* = 0.43), and no significant interactions between task and time (*F*_8,128_ = 0.909, *p* = 0.511) were identified for the test H amplitudes.

We found significant main effects of task (*F*_2,32_ = 13.764, *p* < 0.001) and time (*F*_4,64_ = 3.384, *p* = 0.014) for reciprocal inhibition. A significant interaction between task and time was also observed (*F*_8,128_ = 13.764, *p* = 0.004). *Post hoc* paired *t*-test comparisons showed that reciprocal inhibition was significantly higher at post0 and post10 in the MI + ES group than at baseline (post0: *p* = 0.003, post10: *p* = 0.032; Table 2 and Figure 3C). MI alone and ES alone showed no significant changes in reciprocal inhibition in comparison with baseline at any of the time point evaluated. Reciprocal inhibition in MI + ES was significantly higher than in MI alone at post0 (*p* = 0.007), post10 (*p* = 0.003), and post20 (*p* = 0.028). Reciprocal inhibition in MI + ES was also significantly higher than in ES alone at post0 (*p* = 0.027), post10 (*p* = 0.035), post20 (*p* = 0.026), and post30 (*p* = 0.04).

**Table 2:** Statistical results of the *post hoc* paired *t*-tests with Bonferroni correction in reciprocal inhibition.

Comparison with the baseline within task				
Task	*p*-value [95% confidence interval of mean difference]			
	post0	post10	post20	post30
MI alone	1.0 [-0.079–0.140]	1.0 [-0.170–0.116]	1.0 [-0.086–0.147]	1.0 [-0.070–0.149]
ES alone	1.0 [-0.068–0.103]	1.0 [-0.103–0.067]	1.0 [-0.135–0.096]	0.364 [-0.213–0.037]
MI + ES	0.003^∗∗^ [0.036–0.209]	0.032^∗^ [0.007–0.237]	0.073 [-0.006–0.194]	1.0 [-0.088–0.188]

**Comparison of the value among tasks at the same time points**				
**Task**	***p*-value [95% confidence interval of mean difference]**			
	**post0**	**post10**	**post20**	**post30**

MI alone vs. ES alone	1.0 [-0.068–0.138]	0.624 [-0.059–0.173]	1.0 [-0.094–0.089]	0.062 [-0.163–0.003]
MI alone vs. MI + ES	0.007^∗∗^ [0.032–0.217]	0.003^∗∗^ [0.060–0.302]	0.028^∗^ [0.009–0.182]	0.982 [-0.069–0.153]
ES alone vs. MI + ES	0.027^∗^ [0.009–0.170]	0.035^∗^ [0.008–0.241]	0.026^∗^ [0.011–0.185]	0.04^∗^ [0.005–0.239]


*MI, motor imagery; ES, electrical stimulation; MI + ES, motor imagery combined with electrical stimulation; ^∗∗^p < 0.01, ^∗^p < 0.05.*

### Motor Imagery Ability

The VMIQ-2 mean score was 26.9 ± 8.7 (mean ± SD) for the internal visual imagery subscale, 28.5 ± 11.0 for the external visual imagery subscale, and 26.1 ± 8.4 for the kinesthetic imagery subscale. There were no significant correlations between changes in TA MEPs or reciprocal inhibition after intervention and the VMIQ-2 scores.

## Discussion

We found that ankle dorsiflexion MI combined with ES increased task-related muscle (TA) MEPs and reciprocal inhibition from the TA to the SOL. Intervention had no effect on task-unrelated muscle (SOL) MEPs. MI and ES alone produced no effects on TA or SOL MEPs or reciprocal inhibition. Our study provides the first evidence that a combination of MI and ES is effective at inducing plasticity of both lower limb corticospinal excitability and spinal reciprocal inhibition circuits.

### Corticospinal Excitability

We found that MI + ES resulted in significantly higher TA MEPs immediately after the task than at baseline. These effects remained for up to 10 min after the task. These results indicate that MI combined with ES can induce plastic changes such as long-term potentiation of the primary motor cortex and/or corticospinal circuits (Huang et al., 2011). Our results align with those previously presented by Khaslavskaia and Sinkjaer (2005), where the authors reported that voluntary dorsiflexion combined with ES of the common peroneal nerve maintained the enhanced corticospinal excitability of the TA for longer than voluntary dorsiflexion or ES alone. The combination of the activities of the somatosensory afferents and intrinsic motor cortical circuits may be important for inducing cortical plasticity (Stefan et al., 2000). It is known that MI alone and ES alone (Bakker et al., 2008; Yamaguchi et al., 2012), and MI using electroencephalography combined with ES (Mrachacz-Kersting et al., 2012) increase the excitability of a region of the primary motor cortex in real time. It is possible that the spike-timing-dependent plasticity resulting from combined inputs may have occurred at a synapse somewhere in corticospinal pathway of the TA (Stefan et al., 2000; Stinear and Hornby, 2005). Few studies have investigated the effects of ES alone on MEPs after intervention. One study indicated that ES applied to the common peroneal nerve for 30 min induced plastic changes in TA MEPs (Khaslavskaia and Sinkjaer, 2005). In another study targeting the upper extremity, ES alone for 14 min did not induce any plastic changes (Taylor et al., 2012). Thus, it is possible that the time-window of stimulation used in our study (20 min) was insufficient to induce plastic changes in the corticospinal excitability of the TA following ES. Kaneko et al. (2014) showed that MEP during ES alone was not higher than at rest. The short-term effect of ES alone on corticospinal excitability may be less than that of MI + ES. Yasui et al. (2018) demonstrated that MI during action observation combined with ES significantly increased MEP by 10 min, however, MI during action observation or ES alone for 20 min did not increase MEP. The effect of MI alone for 20 min on corticospinal excitability might be insufficient, just as ES alone.

As expected, we could not find any changes in the task-unrelated muscle (SOL) MEPs following any of our tasks. One previous study examining corticospinal excitability during MI showed that MI increased corticospinal excitability in both task-related and task-unrelated muscles (Bakker et al., 2008). Although we cannot completely discard the possibility of an increase in corticospinal excitability for the task-unrelated muscle given the limited amount of data we were able to collect, we hypothesize that combining ES with MI may help to focalize the effects on excitability in the areas of the primary motor cortex or corticospinal tract related to the task-relevant muscle.

### Reciprocal Inhibition

We found that MI + ES also significantly increased reciprocal inhibition immediately after the task when compared to the baseline. This effect remained up to 10 min after the task. Spinal Ia inhibitory interneurons projecting to the antagonist (SOL) motor neuron receive convergent inputs from the motor cortex and Ia afferents of the agonist muscle (TA) (Nielsen et al., 1993; Masakado et al., 2001). It has been shown that combining descending inputs coming from the corticospinal tracts during voluntary movements with those coming from Ia afferents during ES induces plastic changes of the Ia inhibitory circuit (Yamaguchi et al., 2013; Takahashi et al., 2017, 2018). This mechanism may underlie the effects on reciprocal inhibition we have reported herein. MI and ES alone did not affect reciprocal inhibition. Long-term interventions that target the upper extremities using MI have been known to induce plasticity of the reciprocal inhibition circuits in patients after stroke (Kawakami et al., 2018). However, the effect of short-time MI on reciprocal inhibition remains unknown. Motor cortex excitability is important for maintaining plastic changes in spinal reciprocal inhibition (Wolpaw and Tennissen, 2001; Chen et al., 2006; Fujiwara et al., 2011; Yamaguchi et al., 2013, 2016). Based on our findings, we suggest that short-term motor descending inputs during MI alone are not sufficient to induce plastic changes of the reciprocal inhibition spinal circuits – and that longer times may be needed for the effects to emerge.

In our study, ES alone also did not change reciprocal inhibition. Perez et al. (2003) showed that ES that mimicked aspects of sensory feedback from muscle spindle during walking was effective in inducing plastic changes in reciprocal inhibition, while ES with constant period parameters was ineffective. In our study, stimulus intensity was lower than that found by Perez et al. (2003). The parameters used for the stimulation may be important determinants of the ES effects on the plasticity of the reciprocal inhibition circuits.

### Motor Imagery Ability

Although we first hypothesized that the increase in MEP and reciprocal inhibition after MI + ES would be correlated with an individual’s MI ability, we did not find any significant relationship between the effects on TA MEPs or reciprocal inhibition after MI + ES and the VMIQ-2 scores. MI ability, as assessed by the VMIQ-2, and corticospinal excitability during MI have been known to correlate positively, at least when the upper extremities are the targets for the MI intervention (Lebon et al., 2012; Williams et al., 2012). Previous studies have focused on skillful finger movement imagery. To our knowledge, no previous reports have examined the relationship between MI ability and corticospinal excitability during MI, when the lower limb are the targets for the MI intervention. It is possible that the absence of a correlation between MI + ES effects on MEPs and reciprocal inhibition and MI ability in our study derives from the fact that the movement of the lower limb is coarse when compared with that of skillful finger movement. Future studies examining and comparing different movements will be needed to clarify whether this association is found only in some cases.

### Comparison With Results in the Upper Limb

The present study showed that MI alone and ES alone did not change MEPs or reciprocal inhibition, and that MI + ES effectively increased TA MEP and reciprocal inhibition. The combined effects found were similar to those from a previous study in the upper limb (Yasui et al., 2018). These results suggest that combining ES with MI rehabilitation in the lower limb might be effective for neuromodulation in patients with central nervous system lesions. For gait rehabilitation in patients with central nervous system lesions, some strategies such as robotic gait training (Cheung et al., 2017; Holanda et al., 2017; Bruni et al., 2018) or body-weight support treadmill gait training (Mehrholz et al., 2017) can be used; however, the therapy for lower limb paralysis itself is limited. Our results, which indicated that MI + ES might be an effective treatment of lower limb paralysis, are of clinical importance.

### Clinical Implications

The induction of long-term potentiation in neural circuits is important for motor recovery in patients with central nervous system lesions. Reduced reciprocal inhibition correlates with the development of hyperactive reflexes and spasticity (Crone and Nielsen, 1994; Okuma et al., 2002; Crone et al., 2003). Furthermore, restoring reciprocal inhibition is important to improve the movement of paretic ankles. We suggest that MI + ES is a useful and safe rehabilitation method that can be performed even in patients who are severely paralyzed and have difficulty performing voluntary movements. As a next step, we would like to investigate the effects of MI + ES on reciprocal inhibition in patients with central nervous system lesions.

### Limitations

In this study, we measured the effects of MI + ES in healthy subjects. It is necessary to investigate the effects of MI + ES in patients with central nervous system lesions, i.e., those who would be the target of neuromodulation in clinical practice. We could not measure SOL MEPs from all subjects, which limited our power to detect potential effects on the task-unrelated muscle. A similar problems was faced by Bakker et al. (2008), who was unable to reliably record MEPs of the medial gastrocnemius muscle during ankle dorsiflexion MI. Future studies should consider this limitation when selecting about the MI task and the muscle used to record MEPs.

## Conclusion

Ankle dorsiflexion MI combined with ES enhanced TA MEPs and reciprocal inhibition; these effects persisted after the task. In addition to other descending modulation methods such as brain stimulation or voluntary muscle contraction, the combination of MI with afferent input stimulation through ES may be effective in inducing plasticity of the corticospinal excitability and spinal reciprocal inhibition circuits.

## Author Contributions

YT, MK, and TY contributed to the concept, idea, research design, and project management. YT, MK, TY, and ML wrote the manuscript. YT, YI, and KK recruited participants and collected data. YT and YI performed data analysis. MK, TY, and KK provided facilitates and equipment. ST wrote the program for data collection. ML procured funding. YT, MK, TY, KK, and ML provided consultation (including review of manuscript before submission).

## Conflict of Interest Statement

The authors declare that the research was conducted in the absence of any commercial or financial relationships that could be construed as a potential conflict of interest.
